# 14-3-3 Proteins in Guard Cell Signaling

**DOI:** 10.3389/fpls.2015.01210

**Published:** 2016-01-28

**Authors:** Valérie Cotelle, Nathalie Leonhardt

**Affiliations:** ^1^Laboratoire de Recherche en Sciences Végétales, Université de Toulouse, CNRS, UPSCastanet-Tolosan, France; ^2^UMR7265, Laboratoire de Biologie du Développement des Plantes, Service de Biologie Végétale et de Microbiologie Environnementales, Institut de Biologie Environnementale et Biotechnologie, CNRS–CEA–Université Aix-MarseilleSaint-Paul-lez-Durance, France

**Keywords:** 14-3-3 proteins, guard cell, H^+^-ATPases, ion channels, phototropins, protein kinases, protein phosphorylation, signal transduction

## Abstract

Guard cells are specialized cells located at the leaf surface delimiting pores which control gas exchanges between the plant and the atmosphere. To optimize the CO_2_ uptake necessary for photosynthesis while minimizing water loss, guard cells integrate environmental signals to adjust stomatal aperture. The size of the stomatal pore is regulated by movements of the guard cells driven by variations in their volume and turgor. As guard cells perceive and transduce a wide array of environmental cues, they provide an ideal system to elucidate early events of plant signaling. Reversible protein phosphorylation events are known to play a crucial role in the regulation of stomatal movements. However, in some cases, phosphorylation alone is not sufficient to achieve complete protein regulation, but is necessary to mediate the binding of interactors that modulate protein function. Among the phosphopeptide-binding proteins, the 14-3-3 proteins are the best characterized in plants. The 14-3-3s are found as multiple isoforms in eukaryotes and have been shown to be involved in the regulation of stomatal movements. In this review, we describe the current knowledge about 14-3-3 roles in the regulation of their binding partners in guard cells: receptors, ion pumps, channels, protein kinases, and some of their substrates. Regulation of these targets by 14-3-3 proteins is discussed and related to their function in guard cells during stomatal movements in response to abiotic or biotic stresses.

## Introduction

Reversible protein phosphorylation is recognized as one of the most important post-translational modifications in eukaryotes, playing major roles in the regulation of cellular processes (Cohen, 2002). However, in many cases, phosphorylation alone is not sufficient to achieve complete protein regulation, but is required to induce the binding of interactors that modulate protein function. Among the phosphopeptide-binding proteins, the 14-3-3 proteins are the best characterized in plants (Chevalier et al., 2009). The 14-3-3s form a family of highly conserved proteins found in all eukaryotes that bind to phosphoserine/phosphothreonine-containing motifs. 14-3-3 proteins have been found to be expressed in all eukaryotic organisms, in which they generally exist as multiple isoforms. While yeast expresses two 14-3-3 isoforms and mammals possess seven, plants have a varying number of isoforms, with e.g., thirteen identified in *Arabidopsis* and eight in rice (Van Heusden et al., 1995; DeLille et al., 2001; Aitken, 2006; Yao et al., 2007). *Arabidopsis* 14-3-3 proteins are designated by Greek letters (χ, ω, ѱ, φ, υ, λ, ν, κ, μ, ε, о, ι, π) and are encoded by genes called *General Regulatory Factors* (*GRF1-13*) (Ferl et al., 2002; Chevalier et al., 2009). Both of these designations are currently used for *Arabidopsis* 14-3-3s in the literature. The 14-3-3s are small acidic proteins (∼30 kDa) that are highly conserved both within and across species (Ferl et al., 2002). These proteins form homo- and heterodimers (Paul et al., 2012) that bind, in most cases, to phosphorylated target proteins. To date, three consensus 14-3-3-binding phosphopeptide motifs have been described: mode I (R/K)XX(pS/pT)XP, mode II (R/K)XXX(pS/pT)XP (Muslin et al., 1996; Yaffe et al., 1997) and C-terminal mode III (pS/pT)X_1-2_-COOH (Coblitz et al., 2005; Ganguly et al., 2005), where X is any amino acid and pS/pT represents a phosphoserine or phosphothreonine. However, many phosphorylated target proteins contain 14-3-3-binding sites that do not conform to these consensus motifs and 14-3-3-binding can also occur through non-phosphorylated sequences (Bridges and Moorhead, 2005; Taoka et al., 2011). Through these interactions, 14-3-3s regulate target activity, subcellular localization, proteolysis, or association with other proteins (Cotelle et al., 2000; Taoka et al., 2011; Paul et al., 2012). Plant 14-3-3s interact with a wide range of proteins, thereby playing prominent role in diverse aspects of plant physiology, including primary metabolism, development, abiotic and biotic stress responses, and regulation of stomatal movements (reviewed in Denison et al., 2011; Jaspert et al., 2011; de Boer et al., 2013; Lozano-Durán and Robatzek, 2015).

In plants, the majority of water loss occurs through pores on the leaf surface, which are called stomata. The size of the stomatal pores is variable and controls the rate of diffusion of water vapor out of the plant. In addition to controlling water loss, stomata allow CO_2_ to diffuse into the leaf for photosynthesis. Thus, the primary role of stomata is to optimize the exchange of CO_2_ and water vapor between the intracellular spaces in leaves and the atmosphere according to environmental conditions. Under favorable conditions, stomatal opening requires activation of plasma membrane H^+^-ATPases, resulting in plasma membrane hyperpolarization (Assmann et al., 1985; Shimazaki et al., 1986) to drive K^+^ uptake into guard cells via inward-rectifying K^+^ channels (Schroeder et al., 1984), including K^+^ channels *Arabidopsis thaliana* 1 and 2 (KAT1, KAT2), *Arabidopsis* K^+^ transporter 1 and 2 (AKT1, AKT2) and K^+^ rectifying channel (KC1) (Schachtman et al., 1992; Nakamura et al., 1995; Pilot et al., 2001; Szyroki et al., 2001). Uptake of K^+^ ions, in combination with the accumulation of anions, increases the osmotic potential of the guard cells resulting in guard cell swelling, driving opening of the stomatal pore. In contrast, stomatal closure is triggered by transition from light to darkness, high CO_2_ concentrations and abscisic acid (ABA), a hormone synthesized in response to drought stress. All these signals have been shown to induce an alkalinisation of the apoplastic space, which is correlated with the concomitant decrease of the plasma membrane H^+^-ATPase activity (Hedrich et al., 2001; Jia and Davies, 2006). Moreover, the activation of rapid-type (R-type) anion channels, the aluminum-activated anion channel 12 (ALMT12), and slow-type (S-type) anion channels including slow anion channel-associated 1 (SLAC1) and SLAC1 homolog 3 (SLAH3), facilitate the efflux of anions such as malate^2-^, Cl^-^, and NO_3_^-^ (Schroeder and Hagiwara, 1989; Hedrich et al., 1990; Roelfsema et al., 2004; Negi et al., 2008; Vahisalu et al., 2008; Meyer et al., 2010; Sasaki et al., 2010; Geiger et al., 2011). An elevation of cytoplasmic Ca^2+^ concentration due to the activation of plasma membrane and vacuolar channels is also observed during stomatal closure (Schroeder and Hagiwara, 1989; Ward and Schroeder, 1994). Altogether, inhibition of H^+^-ATPases, activation of anion and Ca^2+^ channels induce plasma membrane depolarization. This plasma membrane depolarization activates guard cell outward-rectifying K^+^ (GORK; Hosy et al., 2003). The efflux of solutes from the guard cells leads to a reduced turgor and stomatal closure.

**FIGURE 1:**
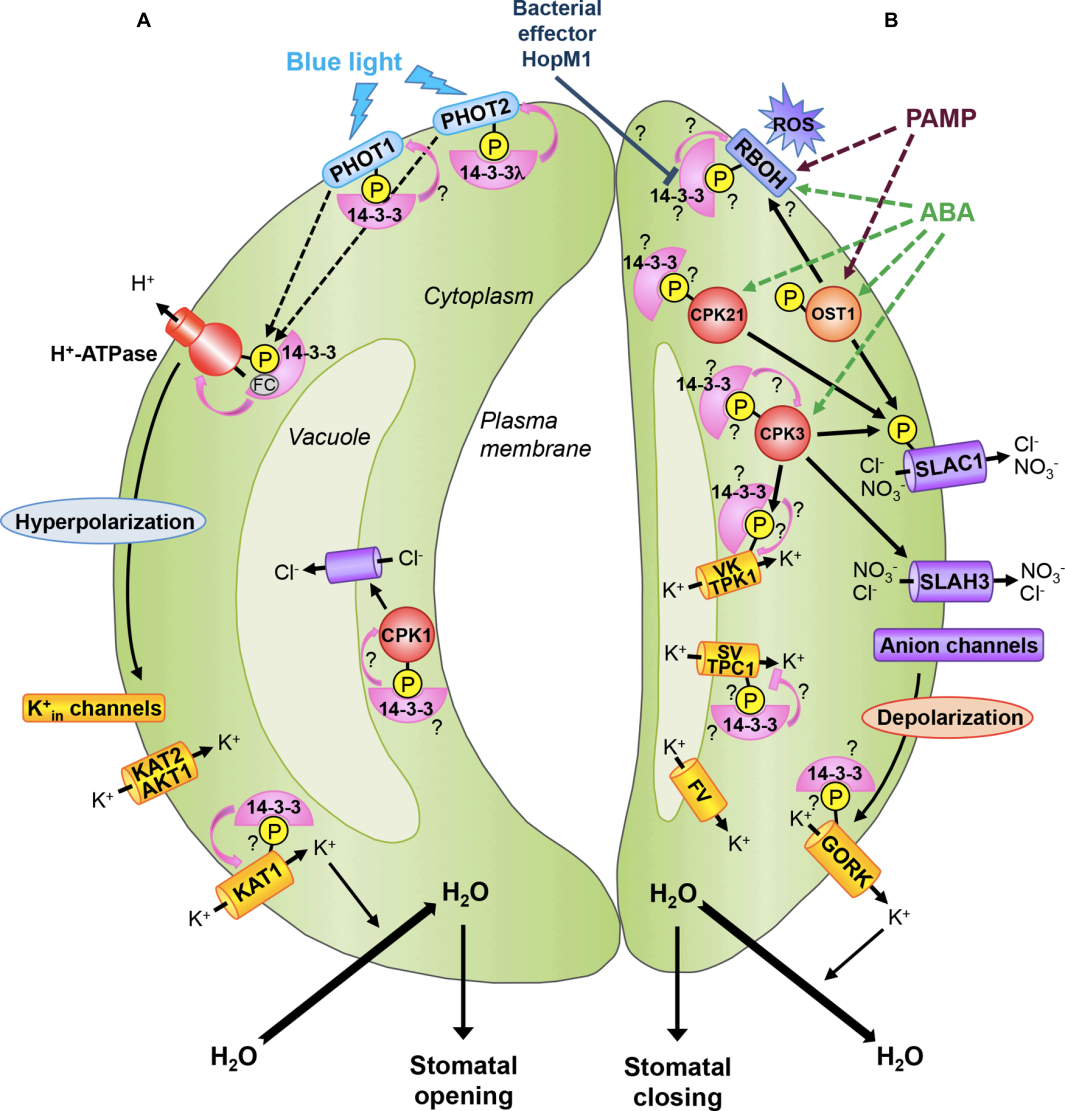
**14-3-3 proteins during stomatal movements.** **(A)** Stomatal opening: the perception of blue light by phototropins PHOT1 and PHOT2 leads to their autophosphorylation and subsequent 14-3-3 binding. In *Arabidopsis*, the 14-3-3λ isoform is required for PHOT2-mediated stomatal opening. Blue light stimulates the plasma membrane H^+^-ATPase by phosphorylation at its C-terminus end and subsequent 14-3-3 binding. The fungal toxin FC stabilizes the 14-3-3/H^+^-ATPase interaction leading to constant activation of the proton pump and thus irreversible stomatal opening. Activation of H^+^-ATPases leads to hyperpolarization of the plasma membrane and uptake of K^+^ via inward-rectifying K^+^ channels (K_in_) including K^+^ channel *A. thaliana* 1 and 2 (KAT1, KAT2) and *Arabidopsis* K^+^ transporter 1 (AKT1). KAT1 is activated by 14-3-3 binding. K^+^ influx induces inward water movement leading to guard cell swelling and stomatal opening. At the tonoplast, a Cl^-^ channel providing a pathway for anion uptake into the vacuole is activated by the calcium-dependent protein kinase (CPK) CPK1, whose activity might be directly stimulated by 14-3-3s. **(B)** Stomatal closing: ABA induces activation of protein kinase open stomata 1 (OST1) as well as CPKs. Among the CPKs involved in guard cell ABA signaling, CPK21 could bind to 14-3-3 proteins and CPK3 might be stabilized by its interaction with 14-3-3s. OST1 and CPKs can activate the guard cell plasma membrane S-type anion channel SLAC1 (slow anion channel-associated 1) by phosphorylation. SLAC1 homolog 3 (SLAH3), another guard cell S-type anion channel, is also activated by CPK3. Activation of anion channels leads to plasma membrane depolarization and activation of the guard cell outward-rectifying K^+^ (GORK) channel which is a putative 14-3-3 client protein. The efflux of ions leads to water loss and guard cell shrinkage, thus closure of the stomatal pore. During stomatal closure, K^+^ release from vacuoles occurs via vacuolar K^+^-selective (VK) channels, slow vacuolar (SV) channels and fast vacuolar (FV) channels. The tandem-pore K^+^ channel 1 (TPK1) represents the guard cell VK channel which might be activated by 14-3-3 binding to a N-terminal site phosphorylated by CPK3. In contrast, SV channels, represented by the two-pore channel 1 (TPC1) in *Arabidopsis*, might be inactivated by 14-3-3 binding. Stomatal closure induced by pathogen-associated molecular patterns (PAMPs) or ABA involves plasma membrane respiratory burst oxidase homologs (RBOHs) which are targets of OST1. RBOHs are NADPH oxidases producing reactive oxygen species (ROS) in the apoplast and might be activated by interaction with 14-3-3 proteins. The *Pseudomonas syringae* effector HopM1 could suppress PAMP-triggered ROS production and stomatal closure by destabilization of 14-3-3 proteins. Pink lines show 14-3-3 regulation on target proteins. Arrowheads designate activation, bars indicate inhibition. Dashed lines denote more than one step, solid lines show direct interaction. Question marks denote signaling events that require further investigation in guard cells. The P in the yellow-colored disks indicates a phosphorylated protein. See the text for details.

In the past decades, guard cell research has revealed many new signal transduction components including channels mediating movement of ions. However, mechanisms by which the environmental cues are transduced to activate or deactivate the channels are still not completely understood. Using several approaches including genetics and biochemistry, the key role of protein phosphorylation involving binding of 14-3-3 proteins has been demonstrated in guard cell signal transduction. Moreover, several studies report expression of 14-3-3 isoforms in guard cells (**Table 1**). In this mini-review, we highlight the functions of 14-3-3 proteins in guard cell signaling, which are summarized in **Figure 1**.

**Table 1:** Expression and subcellular localization of *Arabidopsis thaliana* 14-3-3 proteins.

Gene name	Protein Name	Gene ID	Gene expression	Protein localization	References
*GRF1*	Chi	At4g09000	Seedling; root; root hair; bud; guard cell; flower; anther; stigma; pollen; silique	Cytoplasm; nucleus	^1^Daugherty et al., 1996; ^1,2^Ferl et al., 2002; ^1^Wang et al., 2008; ^1^Zhao et al., 2008; ^1,2^Paul et al., 2012; ^2^Swatek et al., 2014; ^1^Van Kleeff et al., 2014
*GRF2*	Omega	At1g78300	Seedling; root; leaf; stem; flower; pollen; silique; seed	Cytoplasm; nucleus	^2^Cutler et al., 2000; ^1^Sorrell et al., 2003; ^2^Paul et al., 2005; ^1^Schmid et al., 2005; ^1^Wang et al., 2006, 2008; ^1^Hajduch et al., 2010; ^1^Paul et al., 2012; ^2^Yoon and Kieber, 2013
*GRF3*	Psi	At5g38480	Seedling; root; leaf; guard cell; stem; flower; pollen; silique; seed	Cytoplasm; nucleus	^1^Ferl et al., 2002; ^1^Leonhardt et al., 2004; ^1^Schmid et al., 2005; ^1^Wang et al., 2006, 2008; ^1^Rajjou et al., 2008; ^1^Paul et al., 2012; ^1,2^Catalá et al., 2014
*GRF4*	Phi	At1g35160	Root; leaf; guard cell	Plasma membrane; cytoplasm; nucleus; nuclear membrane	^2^Ferl et al., 2002; ^1^Gepstein et al., 2003; ^2^Paul et al., 2005; ^1^Zhao et al., 2008; ^1,2^Paul et al., 2012; ^1^Van Kleeff et al., 2014
*GRF5*	Upsilon	At5g16050	Root; leaf; flower; pollen; silique; seed	Plasma membrane; cytoplasm; nucleus; nuclear membrane; chloroplast	^1,2^Sehnke et al., 2000; ^2^Ferl et al., 2002; ^1^Schmid et al., 2005; ^1^Mayfield et al., 2007; ^1^Wang et al., 2008; ^1^Hajduch et al., 2010; ^2^Pignocchi and Doonan, 2011; ^1^Paul et al., 2012; ^1^Van Kleeff et al., 2014
*GRF6*	Lambda	At5g10450	Seedling; root; leaf; guard cell; stem; flower; silique; seed	Plasma membrane; cytoplasm; nucleus; vacuole	^1,2^Ferl et al., 2002; ^1^Sorrell et al., 2003; ^1^Leonhardt et al., 2004; ^2^Paul et al., 2005; ^1^Schmid et al., 2005; ^2^Latz et al., 2007; ^1^Sullivan et al., 2009; ^1^Hajduch et al., 2010; ^1,2^Paul et al., 2012; ^1,2^Carrasco et al., 2014; ^1^Van Kleeff et al., 2014; ^1,2^Zhou et al., 2014
*GRF7*	Nu	At3g02520	Root; leaf; flower; pollen; silique	Plasma membrane; cytoplasm; nuclear membrane; chloroplast	^1,2^Sehnke et al., 2000; ^2^Ferl et al., 2002; ^1^Schmid et al., 2005; ^1^Wang et al., 2008; ^1^Paul et al., 2012; ^1^Van Kleeff et al., 2014
*GRF8*	Kappa	At5g65430	Seedling; root; leaf; stem; flower; silique; seed	Cell wall; plasma membrane; cytoplasm; nucleus	^2^Ferl et al., 2002; ^1^Sorrell et al., 2003; ^2^Paul et al., 2005; ^1^Schmid et al., 2005; ^1^Hajduch et al., 2010; ^1,2^Paul et al., 2012; ^1^Van Kleeff et al., 2014
*GRF9*	Mu	At2g42590	Seedling; root; leaf; guard cell; stem; flower; silique; seed	Plasma membrane; cytoplasm; nucleus; chloroplast	^1^Kuromori and Yamamoto, 2000; ^1,2^Sehnke et al., 2000; ^1,2^Ferl et al., 2002; ^1^Leonhardt et al., 2004; ^2^Koroleva et al., 2005; ^1^Schmid et al., 2005; ^1^Mayfield et al., 2007; ^1^Hajduch et al., 2010; ^1^Paul et al., 2012; ^2^He et al., 2015
*GRF10*	Epsilon	At1g22300	Root; leaf; flower; pollen; silique; seed	Plasma membrane; cytoplasm; nucleus; nuclear envelope; chloroplast	^1,2^Sehnke et al., 2000; ^1,2^Ferl et al., 2002; ^1^Schmid et al., 2005; ^1^Wang et al., 2008; ^1^Hajduch et al., 2010; ^1,2^Paul et al., 2012; ^2^Swatek et al., 2014


*GRF11*	Omicron	At1g34760	Root; root hair; leaf; guard cell; stem; flower	nd	^1^Rosenquist et al., 2001; ^1^Ferl et al., 2002; ^1^Leonhardt et al., 2004; ^1^Won et al., 2009; ^1^Paul et al., 2012
*GRF12*	Iota	At1g26480	Leaf; flower; pollen	nd	^1^Rosenquist et al., 2001; ^1^Ferl et al., 2002; ^1^Schmid et al., 2005; ^1^Wang et al., 2008; ^1^Paul et al., 2012
*GRF13*	Pi	At1g78220	nd	nd	^1^Paul et al., 2012


**nd: not determined.**

**^1^Reference related to gene expression.**

**^2^Reference related to protein localization.**

## Regulation of Membrane Proteins By 14-3-3 Proteins in Guard Cells

### Phototropins and H^+^-ATPases in Response to Blue Light

Light stimulates stomatal opening via two signaling pathways. One depends specifically on blue light and is perceived by two phototropins, PHOT1 and PHOT2 and cryptochromes, while the other is stimulated by photosynthetically active radiations (Kinoshita et al., 2001; Shimazaki et al., 2007). Phototropins are serine/threonine protein kinases with two LOV (light, oxygen and voltage) domains (Briggs and Christie, 2002). The activated phototropins undergo autophosphorylation and bind 14-3-3 proteins, and ultimately activate the plasma membrane H^+^-ATPase in guard cells (Kinoshita et al., 2001, 2003; Ueno et al., 2005). It is still unknown whether phototropin excitation induces direct phosphorylation of the H^+^-ATPase via a direct association of the two proteins, or whether there are one or more signaling cascade elements. In *Vicia faba* and *A. thaliana* guard cells, blue light has been shown to induce phosphorylation-dependent binding of a non-epsilon 14-3-3 to PHOT1 (Kinoshita et al., 2003; Ueno et al., 2005). Using yeast two-hybrid and *in vitro* assays, PHOT1 was found to interact with 14-3-3λ with the strongest affinity followed by 14-3-3κ, 14-3-3φ, and 14-3-3υ (Sullivan et al., 2009). However, characterization of *Arabidopsis* mutants lacking both 14-3-3λ and 14-3-3κ has been unsuccessful in identifying a physiological role for 14-3-3-binding to PHOT1 (Sullivan et al., 2009). More recently, Tseng et al. (2012) demonstrate that 14-3-3λ interacts also with PHOT2 and plays a role in PHOT2-mediated blue light response. This interaction is dramatically reduced when the PHOT2 S747 in a putative mode I 14-3-3 recognition site was replaced by a non phosphorylatable residue. In addition, blue light-induced stomatal opening is dramatically impaired in *phot1-5 14-3-3λ Arabidopsis* double mutant. In contrast, *phot2-1 14-3-3λ* double mutant and *phot1-5 14-3-3κ* double mutant do not exhibit defects in stomatal opening in response to blue light. Altogether, these observations demonstrate that the closely related 14-3-3 isoforms λ and κ differentially affect PHOT2 signaling in guard cell and reveal the existence of remarkable functional specificity of 14-3-3 proteins.

Furthermore, blue light activates the plasma membrane H^+^-ATPase (Shimazaki et al., 1986; Kinoshita and Shimazaki, 1999; Hayashi et al., 2011). The pump activation requires the phosphorylation of its penultimate threonine residue at the C-terminus end. However, this phosphorylation alone is not enough to activate H^+^ pumping, as subsequent binding of 14-3-3 proteins is also needed (Palmgren, 2001; Bækgaard et al., 2005). In *Vicia faba* guard cells, a 32 kDa 14-3-3 protein has been shown to bind to the phosphorylated C-terminus of the H^+^-ATPase, but not to the non phosphorylated one (Emi et al., 2001; Kinoshita and Shimazaki, 2001). Moreover, blue light increases the amount of bound 14-3-3 protein which is proportional to H^+^-ATPase activity (Kinoshita and Shimazaki, 2002). The binding of 14-3-3 proteins to the autoinhibitory C-terminal domain of the H^+^-ATPase prevents its interaction with the catalytic domain leading to a high-activity state of the pump. The H^+^-ATPase/14-3-3 complex is stabilized by fusicoccin (FC), a fungal phytotoxin (Palmgren, 2001). FC binds to 14-3-3 proteins, thereby increasing the affinity of 14-3-3 proteins for the autoinhibitory C-terminal end of the plasma membrane H^+^-ATPase, which causes irreversible opening of stomata (Assmann and Schwartz, 1992; Kinoshita and Shimazaki, 2001). Interestingly, Pallucca et al. (2014) show that H^+^-ATPase preferentially interacts with non-ε 14-3-3 isoforms. However, further studies will be needed to identify which 14-3-3 isoforms interact with guard cell-expressed proton pumps. Finally, overexpression of 14-3-3λ in cotton results in an increase in stomatal conductance suggesting that 14-3-3λ may interact with the plasma membrane H^+^-ATPase or phototropins to regulate stomatal movements (Yan et al., 2004).

### Ion Channels at the Plasma Membrane

Plasma membrane K^+^ channels play a major role in K^+^ fluxes that modulate guard cell turgor. The main plasma membrane K^+^ channels identified in guard cell are from shaker superfamily (Véry et al., 2014). In *Arabidopsis* guard cells, the expression of six shaker-type K^+^ channels can be detected including KAT1, KAT2, AKT1, AKT2, GORK, and KC1 (Schachtman et al., 1992; Nakamura et al., 1995; Pilot et al., 2001; Szyroki et al., 2001). KAT1, the first cloned plant K^+^ channel, was demonstrated to be endowed with functional properties compatible with a role in mediating K^+^ influx (Schachtman et al., 1992). KAT1 is the main inward-rectifying K^+^ channel in guard cell since its disruption leads to more than 50% reduction of the inward K^+^ currents in *Arabidopsis* guard cell (Szyroki et al., 2001). Moreover, dominant negative repressive mutants of KAT1 and KAT2 suppress light- and low-CO_2_-induced stomatal opening (Kwak et al., 2001; Lebaudy et al., 2008). These data provide genetic evidences demonstrating the important role of inward K^+^ channels for stomatal opening. Other mechanisms are also involved in the regulation of these channel activities. Notably, KAT1 is sensitive to internal and external pH (Blatt, 1992; Hoth et al., 2001). Cytosolic 14-3-3 proteins also regulate KAT1 activity. The binding of the maize GF14-6 isoform to KAT1 enhances channel activity by increasing channel open probability and also by controlling the number of channels at the plasma membrane (Sottocornola et al., 2006, 2008).

In contrast to inward K^+^ channels, only one outward rectifying K^+^ channel, GORK, is expressed in *Arabidopsis* guard cell. GORK disruption completely abolishes outward K^+^ channel currents in guard cells and impairs dark- and ABA-induced stomatal closure (Hosy et al., 2003). GORK currents are regulated by external K^+^ concentration and also by internal and external pH (Blatt, 1992; Ache et al., 2000). Interestingly, by mass spectrometry-based proteomic analysis of tag affinity-purified 14-3-3ω complexes, GORK was identified as a putative 14-3-3 client (Chang et al., 2009). However, further studies will be required to determine the physiological function of GORK regulation by 14-3-3s in guard cells.

### Vacuolar Ion Channels

During stomatal closure, K^+^ release from vacuoles into the cytosol occurs via channels. Three cation channel activities have been characterized in guard cell tonoplast: fast vacuolar (FV) channels, vacuolar K^+^-selective (VK) channels, and slow vacuolar (SV) channels (Pandey et al., 2007). FV channels are instantaneously activated in response to voltage and inhibited by cytosolic Ca^2+^ concentrations ([Ca^2+^]_cyt_) (Hedrich and Neher, 1987; Allen and Sanders, 1996). However, the molecular identity of these channels still remains unknown. VK channels are voltage-independent, K^+^-selective, and activated by increases in [Ca^2+^]_cyt_ (Ward and Schroeder, 1994; Allen and Sanders, 1996). In *Arabidopsis*, the voltage-independent K^+^-channels of the TPK/KCO family consists of five “tandem-pore” channels (TPK1-TPK5) and one K_ir_-like channel (KCO3) (Voelker et al., 2010). Except for TPK4, these channels are located in the tonoplast and contain 14-3-3-binding sites and Ca^2+^-binding EF-hands in their N- and C-termini, respectively (Voelker et al., 2006). Their conserved 14-3-3-binding site conforms to the consensus mode I binding motif with a serine or threonine residue as potential phosphorylation site (Latz et al., 2007). Using this phosphorylated conserved motif in surface plasmon resonance (SPR) experiments, it was demonstrated that HvKCO1/HvTPK1, a barley homologue of *Arabidopsis* TPK1, interacts with three out of the five barley 14-3-3 isoforms (Sinnige et al., 2005). Moreover, *Arabidopsis* TPKs (TPK1, 3 and 5) also bind to 14-3-3s both *in vitro* and *in vivo* (Latz et al., 2007; Voelker et al., 2010; Shin et al., 2011). Phosphorylation of the 14-3-3-binding motif in TPK1 and TPK5 appears to be a prerequisite for their interaction with 14-3-3s. Indeed, in yeast two-hybrid assays, mutating serine or threonine residue to alanine in the 14-3-3-binding sites (TPK1 : S42A; TPK5 : T83A) abolishes the interactions between channel N-terminal segments and 14-3-3s (Voelker et al., 2010). In plant cells, TPK1, but not the TPK1-S42A mutant, co-localizes with 14-3-3λ at the tonoplast (Latz et al., 2007). In the same study, pull-down assays and surface plasmon resonance measurements show high affinity interaction of 14-3-3λ with phosphorylated TPK1. After TPK1 expression in yeast and isolation of vacuoles, 14-3-3λ when applied to the cytosolic side of the membrane, strongly increases TPK1 currents in patch-clamp experiments. TPK1 channel activity in yeast exihibits all the hallmarks of the VK channel, i.e., K^+^ selectivity, activation by cytosolic Ca^2+^, and voltage independence (Bihler et al., 2005; Latz et al., 2007). Furthermore, instantaneous VK channel currents are absent in *tpk1* knockout mutants (Gobert et al., 2007). Based on these results, it is assumed that TPK1 represents the VK channel characterized in guard cells (Ward and Schroeder, 1994; Allen and Sanders, 1996). In accordance, TPK1 loss-of-function mutants display slower ABA-induced stomatal closure, thus providing evidence that VK channels can mediate vacuolar K^+^ efflux for stomatal closing (Gobert et al., 2007). SV channels are cation permeable, voltage-regulated and slowly activated at elevated [Ca^2+^]_cyt_ (Hedrich and Neher, 1987; Ward and Schroeder, 1994; Allen and Sanders, 1996). The SV channels are ubiquitous in plants and encoded by the single *TPC1* (*two-pore channel 1*) gene in *Arabidopsis* (Peiter et al., 2005; Ranf et al., 2008). In *tpc1* knockout mutants, inhibition of stomatal opening by extracellular Ca^2+^ is impaired, whereas ABA-promoted stomatal closure is not affected (Peiter et al., 2005). Besides Ca^2+^, SV channels have also been reported to be regulated by 14-3-3 proteins. Indeed, in mesophyll cell vacuoles, the barley SV channel is strongly inhibited by the barley 14-3-3B isoform and 14-3-3λ suppresses SV channel currents in *Arabidopsis* (Van den Wijngaard et al., 2001; Latz et al., 2007). Interestingly, TPC1 has the C-terminal sequence STSDT, which is a potential 14-3-3 type III binding site (Furuichi et al., 2001). However, although SV and VK channels have been both shown to be regulated by 14-3-3 proteins, further studies are required to address the physiological role of these regulations in stomatal movements.

## Regulation of Protein Kinases and Their Subtrates by 14-3-3 Proteins in Guard Cells

Protein phosphorylation plays key roles in regulation of stomatal movements (Zhang et al., 2014). Among the kinases involved in guard cell signaling, calcium-dependent protein kinases (CDPKs) can act as Ca^2+^ sensors able to translate Ca^2+^ transients into specific phosphorylation events (Boudsocq and Sheen, 2013; Liese and Romeis, 2013). In *Arabidopsis*, the CDPK gene family encompasses 34 members (Cheng et al., 2002). Two *Arabidopsis* CDPK isoforms (also named CPKs), CPK1 and CPK3, regulate ion channels in guard cells and have been identified as 14-3-3 targets. Indeed, three *Arabidopsis* 14-3-3 isoforms, ω, ψ, and φ, stimulate autophosphorylated CPK1 *in vitro* by direct binding and CPK1 interacts with endogenous 14-3-3ω (Camoni et al., 1998; Chang et al., 2009). In guard cells, CPK1 activates a vacuolar Cl^-^ channel which may provide a pathway for anion uptake into the vacuole required for stomatal opening (Pei et al., 1996). Recently, CPK3, previously identified as a 14-3-3-binding protein *in vitro* (Moorhead et al., 1999; Cotelle et al., 2000), was found associated with 14-3-3 proteins in *Arabidopsis* (Lachaud et al., 2013). CPK3 is not activated *in vitro* by 14-3-3 proteins (Moorhead et al., 1999), but its interaction with 14-3-3s protects CPK3 from proteolysis (Cotelle et al., 2000; Lachaud et al., 2013). CPK3 directly interacts with the VK channel TPK1 (see above) at the tonoplast and is able to phosphorylate the 14-3-3-binding motif (S42) in the N-terminus of TPK1 (Latz et al., 2013). Moreover, CPK3 does not only phosphorylate sites mediating 14-3-3 binding and interact with 14-3-3s, but this kinase is also able to phosphorylate 14-3-3 proteins themselves, suggesting a cross-regulation between CPK3 and 14-3-3s (Lachaud et al., 2013; Swatek et al., 2014). CPK3 interaction with 14-3-3 proteins has not been described in guard cells, but CPK3 is one of the CDPKs involved in the activation of anion channels at the plasma membrane of guard cells, which is a critical step in stomatal closure (Mori et al., 2006; Geiger et al., 2010, 2011; Brandt et al., 2012; Scherzer et al., 2012; Brandt et al., 2015). Guard cells of double *cpk3 cpk6* knockout mutants show impaired ABA and Ca^2+^ activation of S-type anion channels, and ABA- and Ca^2+^-induced stomatal closing are also partially inhibited in these mutants (Mori et al., 2006). Furthermore, CPK3 and CPK6 activate guard cell slow anion channels SLAC1 and SLAH3 in *Xenopus* oocytes, and are able to phosphorylate SLAC1 *in vitro* (Brandt et al., 2012; Scherzer et al., 2012). Interestingly, SLAC1 is also activated by CPK21 (Geiger et al., 2010) whose closest homologue in tobacco (*Nicotiana tabacum*), NtCDPK1, is a 14-3-3-binding protein. NtCDPK1 acts as a scaffold transferring 14-3-3 to its substrate, the transcription factor REPRESSION OF SHOOT GROWTH (RSG) after its phosphorylation, thus promoting RSG interaction with 14-3-3 proteins which negatively regulate RSG by sequestering it in the cytoplasm (Ito et al., 2014).

Besides CDPKs, the Ca^2+^-independent protein kinase OST1 (open stomata 1), which plays a central role in stomatal closure (Kollist et al., 2014), could also mediate 14-3-3 binding to partners in guard cells. Indeed, targets of *Arabidopsis* OST1 include KAT1 (Sato et al., 2009), the bZIP transcription factor ABA-responsive-element binding factor 3 (ABF3) (Sirichandra et al., 2010) and plasma membrane respiratory burst oxidase homologs (RBOHs) (Sirichandra et al., 2009; Acharya et al., 2013). RBOHs are NADPH oxidases generating reactive oxygen species (ROS) which are important secondary messengers in stomatal closure induced by ABA or pathogen-associated molecular patterns (PAMPs) (Kwak et al., 2003; Mersmann et al., 2010; Macho et al., 2012). The tobacco 14-3-3 isoform Nt14-3-3h binds the C-terminus of the tobacco NADPH oxidase NtrbohD in yeast (Elmayan et al., 2007) and it has been speculated that the *Pseudomonas syringae* effector HopM1, which significantly contributes to bacterial pathogenesis, suppresses PAMP-triggered ROS production and stomatal closure through degradation of 14-3-3κ in *Arabidopsis* (Lozano-Durán et al., 2014). Moreover, in *Vicia faba*, an ortholog of OST1, AAPK (ABA-activated protein kinase), is able to phosphorylate a 61 kDa protein whose binding to a 14-3-3 protein is induced by ABA in guard cells (Takahashi et al., 2007).

## Conclusion and Future Perspectives

Although many indirect indications point out that 14-3-3 proteins play important roles in stomatal movements, regulation of target proteins by 14-3-3s has been characterized in only a few cases in guard cells, as described in this mini-review. Therefore, many questions remain to be addressed in guard cells. What is the extent of the 14-3-3 interactome? What is the functional consequence of 14-3-3 binding to targets and how are these interactions regulated? What is the specificity of 14-3-3 isoforms towards their targets and in the regulation of stomatal movements? Combining protein biochemistry, cell biology and genetics approaches, future work addressing these questions will further our knowledge with regard to the role of 14-3-3 proteins in guard cell signaling.

## Author Contributions

VC and NL contributed equally to the writing and editing of the manuscript.

## Conflict of Interest Statement

The authors declare that the research was conducted in the absence of any commercial or financial relationships that could be construed as a potential conflict of interest.
